# Supplementary material for: Analysis of PPARγ Signaling Activity in Psoriasis
Source: Int J Mol Sci. 2021 Aug 10;22(16):8603. doi: 10.3390/ijms22168603 (PMC8395241; doi:10.3390/ijms22168603)
Supplement: Supplementary file 1 [file ijms-22-08603-s001.zip › Supplemental materials_Analysis of PPARg signaling activity in psoriasis/Pathway models/Models images and html files/Model of PPARG signaling in psoriasis/43661.html]

MAVS


# Protein MAVS

|  |  |
| --- | --- |
| URN | urn:agi-llid:57506 |
| Total Entities | 0 |
| Connectivity | 1018 |
| Name | MAVS |
| Description | mitochondrial antiviral signaling protein |
| Notes | This gene encodes an intermediary protein necessary in the virus-triggered beta interferon signaling pathways. It is required for activation of transcription factors which regulate expression of beta interferon and contributes to antiviral immunity. Multiple transcript variants encoding different isoforms have been found for this gene. [provided by RefSeq, Sep 2011] |
| ObjectType | Protein |

---

|  |  |
| --- | --- |
| Pathway | NOD-like Receptors |
|  | Antiviral Signaling through Pattern Recognition Receptors |
|  | TLR4 Signaling in Leukocytes |
|  | Toll-like Receptors in Sterile Inflammation |
|  | MAVS in Antiviral Innate Immune Response in Myocarditis |
|  | Dendritic Cell Activation in Systemic Lupus Erythematosis |
|  | Crohn's Disease |
|  | Macrophage Dysfunction |
|  | Dendritic Cell Dysfunction in Crohn's disease |
|  | pathway 4 |
|  | Model of PPARG signaling in psoriasis |
|  | sars-cov no binding |
|  | dsRNA induces innate immunity and INFs in alveolar cells |
|  | Dendritic Cell Activation in COVID19 |
|  | 1\_Insufficient activation of immune response in the middle ear epithelium cells in otitis media |
|  | 1\_Defects in response to pathogens in the gut promotes inflammation in Crohn's disease |
|  | 3\_Autophagy dysfunction in Crohn's disease |

---

|  |  |
| --- | --- |
| Group | other partners for SARS |

---

|  |  |
| --- | --- |
| MedScan ID | 57506 |

---

|  |  |
| --- | --- |
| LocusLink ID | 57506 |
|  | 228607 |
|  | 311430 |

---

|  |  |
| --- | --- |
| Alias | virus-induced signaling adaptor variant 1a |
|  | interferon-beta promoter stimulator protein 1 |
|  | D430028G21Rik |
|  | CARD adaptor inducing interferon-beta |
|  | CARD adaptor inducing IFN-beta |
|  | mavs |
|  | mitochondrial antiviral signaling protein |
|  | MGC3260 |
|  | VISA |
|  | Cardif |
|  | DKFZp547C224 |
|  | IFN-beta promoter stimulator-1 |
|  | OTTMUSP00000017159 |
|  | FLJ41962 |
|  | MGC25836 |
|  | OTTHUMP00000030141 |
|  | ips-1 |
|  | OTTMUSP00000017160 |
|  | IFN-B promoter stimulator 1 |
|  | RP23-387C21.3 |
|  | virus-induced signaling adaptor variant 1b |
|  | D430028G21 |
|  | mitochondrial antiviral-signaling protein |
|  | mitochondrial anti-viral signaling protein |
|  | FLJ35386 |
|  | KIAA1271 |
|  | FLJ38051 |
|  | MGC93707 |
|  | DKFZp666M015 |
|  | virus-induced signaling adaptor |
|  | FLJ27482 |
|  | IPS1 |
|  | CARD adapter inducing interferon beta |
|  | interferon beta promoter stimulator protein 1 |
|  | putative NF-kappa-B-activating protein 031N |
|  | virus-induced-signaling adapter |
|  | virus-induced signaling adapter |
|  | virus-induced signaling adapter variant 1a |
|  | virus-induced signaling adapter variant 1b |
|  | CARD adapter inducing interferon-beta |

---

|  |  |
| --- | --- |
| GO ID | 0050700 |
|  | 0019901 |
|  | 0035591 |
|  | 0002218 |
|  | 0071360 |
|  | 0042742 |
|  | 0051607 |
|  | 0045087 |
|  | 0032480 |
|  | 0045071 |
|  | 0051091 |
|  | 0043123 |
|  | 0071660 |
|  | 0071651 |
|  | 0002230 |
|  | 0032727 |
|  | 1902741 |
|  | 0032728 |
|  | 0035549 |
|  | 2000778 |
|  | 0032757 |
|  | 0002735 |
|  | 0042307 |
|  | 0001934 |
|  | 0060760 |
|  | 0045944 |
|  | 0032760 |
|  | 1904469 |
|  | 0060340 |
|  | 1900063 |
|  | 0006355 |
|  | 0007165 |
|  | 0016032 |
|  | 0016021 |
|  | 0031966 |
|  | 0005741 |
|  | 0005739 |
|  | 0005778 |
|  | 0039529 |
|  | 0002376 |
|  | 0032481 |
|  | 0016020 |
|  | 0005777 |
|  | 0051092 |
|  | 0005622 |
|  | 0005515 |
|  | 0033160 |
|  | 0042993 |
|  | 0004871 |
|  | 0005737 |
|  | 0019048 |

---

|  |  |
| --- | --- |
| KEGG ID | hsa:57506 |
|  | mmu:228607 |
|  | rno:311430 |

---

|  |  |
| --- | --- |
| Organism | Homo sapiens {Organism urn:agi-taxid:9606} |
|  | Mus musculus {Organism urn:agi-taxid:10090} |
|  | Rattus norvegicus {Organism urn:agi-taxid:10116} |
|  | Homo sapiens |
|  | Mus musculus |
|  | Rattus norvegicus |

---

|  |  |
| --- | --- |
| Mouse chromosome position | 2 |

---

|  |  |
| --- | --- |
| OMIM ID | 609676 |

---

|  |  |
| --- | --- |
| Rat chromosome position | 3q36 |

---

|  |  |
| --- | --- |
| Hugo ID | 29233 |
|  | HGNC:29233 |

---

|  |  |
| --- | --- |
| Human chromosome position | 20p13 |

---

|  |  |
| --- | --- |
| Swiss-Prot Accession | Q7Z434 |
|  | Q7Z434.2 |
|  | Q3U5B1 |
|  | Q8VCF0 |
|  | Q8VCF0.1 |
|  | Q66HG9 |
|  | Q66HG9.1 |
|  | A8K6X0 |
|  | B2BD33 |
|  | B2BD34 |
|  | F5H6C8 |
|  | M1P2Z0 |
|  | Q2HWT5 |
|  | Q3I0Y2 |
|  | Q5T7I6 |
|  | Q86VY7 |
|  | Q9H1H3 |
|  | Q9H4Y1 |
|  | Q9H8D3 |
|  | Q9ULE9 |
|  | A2ANC9 |

---

|  |  |
| --- | --- |
| GenBank ID | NC\_000020 |
|  | NR\_037921 |
|  | NM\_001206491 |
|  | NP\_001193420 |
|  | NM\_020746 |
|  | NP\_065797 |
|  | NG\_030028 |
|  | AL109804 |
|  | AL353194 |
|  | CH471133 |
|  | EAX10481 |
|  | EAX10482 |
|  | CQ871280 |
|  | CAH56821 |
|  | AB033097 |
|  | BAA86585 |
|  | AB097003 |
|  | BAC77356 |
|  | AB232371 |
|  | BAE79738 |
|  | AK023799 |
|  | BAB14684 |
|  | AK056260 |
|  | AK092705 |
|  | AK095370 |
|  | AK123956 |
|  | BAC85734 |
|  | AK130992 |
|  | BAC85473 |
|  | AK291785 |
|  | BAF84474 |
|  | AK296897 |
|  | BAG59455 |
|  | AL390136 |
|  | AL833736 |
|  | BC000073 |
|  | BC028962 |
|  | BC040719 |
|  | BC040930 |
|  | BC044952 |
|  | AAH44952 |
|  | BC052583 |
|  | BC062310 |
|  | BC063593 |
|  | BM834250 |
|  | BX649084 |
|  | DB219666 |
|  | DQ167126 |
|  | ABA54890 |
|  | DQ174270 |
|  | AAZ80417 |
|  | DQ181928 |
|  | ABA19229 |
|  | EF467323 |
|  | ABR24161 |
|  | EF467324 |
|  | ABR24162 |
|  | KC415005 |
|  | AGF94754 |
|  | Q7Z434 |
|  | NC\_000068 |
|  | NM\_144888 |
|  | NP\_659137 |
|  | NM\_001206382 |
|  | NP\_001193311 |
|  | NM\_001206385 |
|  | NP\_001193314 |
|  | NM\_001206383 |
|  | NP\_001193312 |
|  | AL808128 |
|  | CH466519 |
|  | EDL28315 |
|  | EDL28316 |
|  | AK028421 |
|  | BAC25940 |
|  | AK028708 |
|  | AK085038 |
|  | AK153748 |
|  | BAE32168 |
|  | AK161823 |
|  | AK177575 |
|  | AK178965 |
|  | AK181725 |
|  | AK189538 |
|  | AK193001 |
|  | AK195306 |
|  | AK203321 |
|  | AX775773 |
|  | CAE11586 |
|  | BC020006 |
|  | AAH20006 |
|  | BC025825 |
|  | AAH25825 |
|  | BC031352 |
|  | AAH31352 |
|  | BC037391 |
|  | AAH37391 |
|  | DQ167127 |
|  | ABA54891 |
|  | DQ174271 |
|  | AAZ80418 |
|  | Q8VCF0 |
|  | NC\_005102 |
|  | XM\_006235034 |
|  | XP\_006235096 |
|  | XM\_006235036 |
|  | XP\_006235098 |
|  | XM\_008762179 |
|  | XP\_008760401 |
|  | XM\_006235035 |
|  | XP\_006235097 |
|  | XM\_006235037 |
|  | XP\_006235099 |
|  | NM\_001005556 |
|  | NP\_001005556 |
|  | AC\_000071 |
|  | AAHX01025077 |
|  | AC105811 |
|  | AC110439 |
|  | CH473949 |
|  | EDL80235 |
|  | BC081869 |
|  | AAH81869 |
|  | Q66HG9 |
|  | XP\_008760402 |
|  | XM\_008762180 |
|  | EU832500 |
|  | EU832581 |
|  | CAI18851 |
|  | CAM22812 |
|  | AC\_000152 |
|  | CAI11041 |
|  | AABR06026197 |
|  | NC\_018931 |
|  | AAHY01020738 |
|  | AMYH02036611 |
|  | AC\_000024 |
|  | AMYH02036609 |
|  | AMYH02036610 |
|  | AAHY01020735 |
|  | AAHY01020736 |
|  | AAHY01020737 |
|  | ABBA01053838 |
|  | ACE86983 |
|  | ACE87672 |
|  | ABBA01053835 |
|  | ABBA01053837 |
|  | ABBA01053836 |

---

|  |  |
| --- | --- |
| Swiss-Prot ID | MAVS\_HUMAN |
|  | MAVS\_MOUSE |
|  | MAVS\_RAT |

---

|  |  |
| --- | --- |
| Cell Localization | Mitochondrion outer membrane |
|  | Mitochondrion |
|  | Peroxisome |

---

|  |  |
| --- | --- |
| Ensembl ID | ENSG00000088888 |
|  | ENSP00000413749.2 |
|  | ENST00000416600.6 |
|  | ENSP00000401980.2 |
|  | ENST00000428216.4 |
|  | ENSMUSG00000037523 |
|  | ENSMUSP00000038339.5 |
|  | ENSMUST00000041362.11 |
|  | ENSMUSP00000105828.2 |
|  | ENSMUST00000110199.2 |
|  | ENSRNOG00000025295 |
|  | ENSRNOP00000033476.3 |
|  | ENSRNOT00000034146.3 |
|  | ENSRNOP00000073053.1 |
|  | ENSRNOT00000090764.1 |
|  | ENST00000416600 |
|  | ENSRNOT00000034146 |
|  | ENSRNOT00000090764 |
|  | ENSP00000401980 |
|  | ENSP00000413749 |
|  | ENST00000428216.3 |
|  | ENSMUSP00000038339 |
|  | ENSMUST00000041362 |
|  | ENST00000428216 |
|  | ENSMUST00000110199 |
|  | ENSMUSP00000105828 |
|  | ENSRNOP00000033476 |
|  | ENSRNOP00000073053 |

---

|  |  |
| --- | --- |
| MGI ID | MGI:2444773 |
|  | 2444773 |

---

|  |  |
| --- | --- |
| RGD ID | 1359371 |

---

|  |  |
| --- | --- |
| Unigene ID | Rn.34996 |
|  | Mm.287226 |
|  | Hs.570362 |

---

|  |  |
| --- | --- |
| Homologene ID | 17004 |

---

|  |  |
| --- | --- |
| IPI ID | IPI00020719 |
|  | IPI00411577 |
|  | IPI00411576 |
|  | IPI00122075 |
|  | IPI00364200 |

---
